# *Cystathione gamma lyase*/Hydrogen Sulphide Pathway Up Regulation Enhances the Responsiveness of α1A and α1B-Adrenoreceptors in the Kidney of Rats with Left Ventricular Hypertrophy

**DOI:** 10.1371/journal.pone.0154995

**Published:** 2016-05-18

**Authors:** Ashfaq Ahmad, Munavvar A. Sattar, Maleeha Azam, Mohammed H. Abdulla, Safia A. Khan, Fayyaz Hashmi, Nor A. Abdullah, Edward J. Johns

**Affiliations:** 1 School of Pharmaceutical Sciences, Universiti Sains Malaysia, Penang, 11800, Malaysia; 2 Department of Biosciences, COMSATS Institute of Information Technology, Islamabad, Pakistan; 3 Department of Physiology, University College Cork, Cork, Ireland; 4 Department of Pharmacology, Faculty of Medicine, University of Malaya, Kuala Lumpur, Malaysia; Max-Delbrück Center for Molecular Medicine (MDC), GERMANY

## Abstract

The purpose of the present study was to investigate the interaction between H_2_S and NO (nitric oxide) in the kidney and to evaluate its impact on the functional contribution of α_1A_ and α_1B_-adrenoreceptors subtypes mediating the renal vasoconstriction in the kidney of rats with left ventricular hypertrophy (LVH). In rats the LVH induction was by isoprenaline administration and caffeine in the drinking water together with intraperitoneal administration of H_2_S. The responsiveness of α_1A_ and α_1B_ to exogenous noradrenaline, phenylephrine and methoxaminein the absence and presence of 5-methylurapidil (5-MeU) and chloroethylclonidine (CEC) was studied. *Cystathione gamma lyase* (CSE), cystathione *β synthase* (CBS), *3-mercaptopyruvate sulphar transferase* (3-MST) and endothelial nitric oxide synthase (eNOS) were quantified. There was significant up regulation of CSE and eNOS in the LVH-H_2_S compared to the LVH group (P<0.05). Baseline renal cortical blood perfusion (RCBP) was increased (P<0.05) in the LVH-H_2_S compared to the LVH group. The responsiveness of α_1A_-adrenergic receptors to adrenergic agonists was increased (P<0.05) after administration of low dose 5-Methylurapidil in the LVH-H_2_S group while α_1B_-adrenergic receptors responsiveness to adrenergic agonists were increased (P<0.05) by both low and high dose chloroethylclonidine in the LVH-H_2_S group. Treatment of LVH with H_2_S resulted in up-regulation of CSE/H_2_S, CBS, and 3-MST and eNOS/NO/cGMP pathways in the kidney. These up regulation of CSE/H_2_S, CBS, and 3-MST and eNOS/NO/cGMP pathways enhanced the responsiveness of α_1A_ and α_1B_-adrenoreceptors subtypes to adrenergic agonists in LVH-H_2_S. These findings indicate an important role for H_2_S in modulating deranged signalling in the renal vasculature resulting from LVH development.

## Introduction

Left ventricular hypertrophy (LVH) is characterized by adrenoreceptor over stimulation and sympatho-excitation. The levels of circulating noradrenaline and mean discharge frequency in peripheral sympathetic nerves [1–3] have been reported to be elevated in hypertensive LVH patients. The degree of increased sympathetic activity is proportional to the mass of LV in LVH [4]. Interestingly, renal denervation using the percutaneous technique results in a regression in heart mass and function in LVH patients with a sympatho-inhibition [5]. The hyper-sympathetic activity in LVH is associated with vascular dysfunction and impairment of α_1_-adrenoceptor-mediated renal vasoconstriction[6]. The association of α_1_-adrenoceptors with sympathetic hyperactivity has also been observed in other physiological and pathological states[7,8].

Hydrogen sulphide (H_2_S) is an endothelial derived relaxing factor (EDRF)[9], which is produced endogenously from two sulphur containing amino acids, L-cysteine and L-methionine by the enzymes *cystathionine γ lyse* (CSE) and *cystathionine β synthase* (CBS) [10,11] and acts on K_ATPase_ channels [12]. In mice H_2_S is produced in proximal tubules of kidney [13–15], endothelial cells [16] and vascular smooth muscle [12]. Recently enzymes like 3 MST(3-mercaptopyruvate sulphar transferase) along with cystein amino transferase (CAT), which is similar to aspartate amino transferase [17] have also been observed to produce H_2_S in brain [18]. Recent studies have shown expression of CSE, CBS and 3-MST enzymes in the kidney [19]. H_2_S provides renal protection under ischemia reperfusion injury [20], chronic renal failure [21] and also plays an important role in controlling renal tubular and vascular functions [22]. We previously reported blunt responses of α_1D-_adrenoreceptors to adrenergic stimuli in the kidney of rats with LVH [23], and association of down regulation of CSE and eNOS with decreased responsiveness of α_1A-_adrenoreceptors to adrenergic stimuli in the kidney [24]. Gases like NO and H_2_S have important roles in normal physiological states as well as in diseases and also have an interdependent production [25–29]. H_2_S has been observed to be responsible for NO production in smooth muscles [30,31], while others have shown that NO enhanced the up regulation of H_2_S production in the plasma [32,33].

Despite extensive research on the therapeutic potential of H_2_S in the renal vasculature, potential interactions of H_2_S with α_1_-adrenoceptors under normal physiological conditions and in LVH state remained unexplored. Therefore in the present study we tested the hypothesis that "CSE/H_2_S, CBS, 3-MST and eNOS/NO pathways are down regulated in the kidney of LVH rats and responsible for the blunted responses of α_1A_-adrenoreceptors to adrenergic stimuli." We further hypothesized that "up-regulation of the CSE/H_2_S, CBS and 3-MST pathway by exogenous administration of H_2_S would increase the renal vascular responsiveness to α_1A_ and α_1B_-adrenoceptor activation in the kidneys of LVH rats," in addition" exogenous administration of H_2_S would not only up-regulate the CSE/H_2_S pathway but also will modulate the eNOS/NO/cGMP pathway in the kidney to increase the sensitivity of the α_1A_ and α_1B_-adrenoceptorsby augmentation of responses to adrenergic stimuli."

## Materials and Methods

### Study groups and methodology

The study had been approved by the Animal Research and Service Centre (ARASC) under the Animal Ethics Committee, Universiti Sains Malaysia (AECUSM) with approval no./2012/(76)(364). Male Wistar-Kyoto (WKY) rats (body wt.200±10g) were obtained from the animal house of Universiti Sains Malaysia and given free access to tap water and standard chow (Gold Coin Sdn. Bhd., Penang, Malaysia). Animals were divided into two main groups; one for renal functional study and another for molecular expression studies. One main group consisted of 8 subgroups for functional study of α_1_-adrenoceptors subtypes. The renal functional study group consisted of Control-5MeU, LVH-5MeU, Control-H_2_S+5MeU and LVH-H_2_S+5MeU groups for α_1A_-adrenoceptor evaluation while there were Control-CEC, LVH-CEC, Control-H_2_S+CEC and LVH-H_2_S+CEC (n = 6) for assessment of α_1B_-adrenoceptor functionality. Similarly, the molecular study groups, consisted of Control, LVH, Control-H_2_S and LVH-H_2_S for quantification of CSE and eNOS mRNA expression (3 animals recruited in each group and each animal had triplicate therefore total n = 9 for one group), where by renal cortical tissue was taken for measurement of CSE and eNOS mRNA expression.

LVH was induced by a modification of an earlier model [34] using 5 injections of isoprenaline (5mg/kg s.c) on days 1, 4, 7, 10 and 13 respectively, while caffeine was given in the drinking water (62mg/L) for the 2 weeks time as reported from the same lab [23]. The control group were given 5 saline injections at similar intervals as in the LVH group. H_2_S treatment involved the administration of intraperitoneal NaHS (56μM) daily for 5 weeks [35], beginning three weeks prior to the isoprenaline and caffeine administration.

### Expression profiling of CSE, CBS, 3-MSTand eNOS of H_2_S treated control and LVH rats’ kidney

Molecular study was conducted as described previously [36]. Briefly the protocol consisted of the following steps; after cervical dislocation of the rat, kidney cortex was immediately preserved in RNAlater® Solution (Ambion, Life technologies, USA), while RNaseZap® (ambion, Life technologies, USA) was used to prevent any contamination. TRIzole reagent (Ambion, Life technologies, USA) was used to extract total RNA as per manufacturer guidelines. After homogenization, washing and elution, total RNA was extracted, optimized and quantified for purity and yield respectively using a microplate reader (Bio Tek Instrument. Inc., VT, USA). Total RNA was converted to cDNA by High Capacity RNA-to-cDNA kit (Applied Biosystems™, USA), using Step One Plus RT-PCR (Applied Biosystems, Singapore).

The TaqMan primers and probes (TaqMan^**®**^-Gene Expression assays (Applied Biosystems, USA) used were as follows: (1) *CSE* (Gen Bank accession No. NM_017074.1 and Rn00567128_m1) gene [37]; (2) *CBS* (Gen Bank accession No. NM_012522.2 and Rn00560948_m1) gene [19]; (3) *3-MST* (Gen Bank accession No. NM_138843.1 and Rn00593744_m1) gene [19]; (4) *eNOS* (Gen Bank accession No. NM_021838.2 and Rn02132634_s1) gene [38,39]; (5) *β-actin* (Gen Bank accession No. NM_031144.2 and Rn00667869_m1) gene [40,41].

Quantitative RT-PCR reactions were carried out on 3 experimental animals of one group (3x4 = 12 animals), while each rat was further analysed in triplicate using kidney cortex. Beta actin was used as an internal control. The relative quantification of target gene CSE and beta actin, comparative C_T_ (threshold cycle) method with arithmetic formula (2^-ΔΔCT^) was applied [42].

### CSE activity in cardiac tissue of control, LVH, control-H_2_S and LVH-H_2_S groups

Kidney tissue CSE activity was measured by a method described previously [10,43]. Briefly the protocol consisted of homogenization of kidney tissue in 50mmol/L ice cold potassium phosphate buffer (pH 6.8). The reaction mixture consisted of 100 mmol/L of potassium phosphate buffer (pH 7.4), 10mmol/L of L-cysteine, 2mmol/L of pyridoxal 5-phosphate and 10% w/v of cardiac tissue. Cryo vial tubes each containing 0.5ml of 1% zinc acetate were used as centre wells to trap the gas. An Erlenmeyer Pyrex flask (25 ml volume) was used for the reaction. Both, the flask containing reaction mixture and centre wells were flushed with N_2_ and were sealed with paraffin film. The reaction was carried out by initially transferring the reaction flask from ice to shaking water bath at 37°C. After incubation for 90 minutes 0.5 ml of 50% trichloroacetic acid was added to the reaction mixture to stop the reaction. Flask was sealed again and incubated at 37°C for 60 minutes to make sure the complete trapping of H_2_S released from the reaction mixture. The contents of centre wells were transferred to test tubes each containing 3.5 ml of water. Afterward, 0.5 ml of 20 mmol/L of N, N-2 dimethyl-p-phenylenediamine, sulphate in 7.2 mol/L of HCL was added, followed by addition of 0.4 ml of 30 mmol/L of FeCL_3_ in 1.2 mol/L HCL. Absorbance of the resultant reaction mixture was taken at 670nm. H_2_S concentration was measured by using standard curve of H_2_S solutions (3.125–100μM).

### H_2_S measurement in kidney and urine

The H_2_S measurement in kidney tissue was adapted from a previous report [22]. Briefly, renal tissue (50 mg) was homogenized in 0.5 ml of 1% zinc acetate and mixed with 0.5 ml of borate buffer (pH 10.01). After this, a volume of 0.5 ml of N, N-2 dimethyl -p-phenylenediamine (20mM) and 0.5 ml of 300mM FeCL_3_ were added to the tissue homogenate. Reaction tubes were immediately sealed and incubated for 30 minutes with shaking at 37°C. After incubation, all the samples were centrifuged and absorbance of resultant supernatant layer was measured at 670 nm. H_2_S concentration was measured by constructing the standard curve by using known concentrations (3.125–100μM) of NaHS as standard. Concentration of H_2_S in the urine was also measured by following the same method reported for plasma H_2_S measurement [35].

### NO and cGMP level measurements in kidney

The concentration of nitric oxide (nitrite/nitrate) in tissues was determined using a laboratory kit (NJJC Bio Inc., Nanjing, China) following manufacturer's protocol. The cGMP measurements were done using cGMP Direct Immunoassay Kit (Abcam). The main steps during the procedure involved sample preparation, standard curve preparation, acetylation was optional one but it was performed, followed by quantification of cGMP and measurement of optical density at 450 nm.

### Agonists and antagonists used in experiment

The present study used 3 agonists noradrenaline (NA), phenylephrine (PE) and methoxamine (ME). Noradrenaline (NA, Sanofi Winthrop, Surrey, UK) is a non-selective α adrenergic agonist which acts on α_1_ and α_2_ adrenergic receptor; methoxamine (ME, Wellcome, London, UK) is a relatively selective agonist for α_1A_ adrenoreceptors [7,44]; phenylephrine (PE, Knoll, Nottingham, UK) has the ability to act non-selectively on α_1A_, α_1B_ and α_1D_ adrenoceptors [7].

Present study used 2 adrenergic antagonists 5-methylurapidil and choloroethylclonidine (CEC). The 5 methylurapidil (Research Biochemicals International, Natick, MA, USA) is a relatively selective antagonist for the α_1A_-adrenoceptor subtype [45]; chloroethylclondine (Research Biochemicals International, Natick, MA, USA) is a relatively selective antagonist for the α_1B_-adrenoreceptor subtype [46]. Sodium chloride (Sigma-Aldrich, UK).

### Acute experiment

Overnight fasted rats were anesthetized using 60mg/kg pentaobarbitone intraperitoneally (Nembutal; CEVA Sante Animale, Libourne, France). Tracheotomy was performed to facilitate the breathing throughout the experiment. The carotid artery was cannulated (Portex, Kent, UK) and the cannula was attached to a pressure transducer (Gould P23 ID; Statham Instruments) connected to a PowerLab data acquisition system for continuous monitoring of systemic hemodynamics. The left jugular vein was also cannulated (Portex, Kent, UK) to permit the infusion of maintenance doses of anesthesia when required. The left kidney was exposed through an abdominal incision and a laser Doppler flow probe (ADInstrument) was placed superficially onto the surface of the kidney cortex for the renal cortical blood perfusion measurements [47]. The iliac artery was cannulated (Portex, Kent, UK) by inserting the cannula up to the level of the renal artery in such a way that adrenergic agonists were directly delivered to the renal artery [35,48,49]. The animals were allowed a stabilization period of at least 1 hour before the onset of vasoconstrictor experiment. During this period of stabilization mean arterial pressure (MAP), systolic blood pressure (SBP) were measured as LVH markers. Later at the end of experiment and LV index was measured to observe the induction of LVH and effect of H_2_S on the regression of LVH.

### Acute vasoconstrictor study

The acute renal vasoconstrictor study was performed following the procedure reported earlier [48–51]. NA, PE and ME were infused intra-renally in increasing and decreasing dosage levels in such a way that net response was calculated as the average of the increasing and decreasing doses. NA was given in 25, 50, 100 and 200 ηg; PE was administered as 0.25, 0.50, 1 and 2 μg and ME was administered as 0.25, 0.50, 1 and 2 μg [7,23,50]. Fresh solutions were prepared daily. Experiments were divided into 3 phases consisting of a saline phase, low dose antagonist phase and high dose antagonist phase. In the saline phase, saline was infused into the kidney at a rate of 6ml/kg/h; in the low dose phase the antagonists were given as a bolus dose 5μg/kg followed by a maintenance dose of 1/4^th^ the bolus dose per h (MeU and CEC bolus dose of 5μg/kg and 10μg/kg intra-renally followed by 1.5μg/kg/h and 1.5μg/kg/h; CEC at 5mg/kg followed by 1.25mg/kg/h) during the ascending and descending doses of adrenergic agonists.

### Histopathology of control, LVH, control-H_2_S and LVH-H_2_S rat kidney

The right kidney was extracted and preserved in 10% formalin for histopathology study following embedding, trimming and sectioning and LV tissue underwent staining with hematoxyllin and eosin staining [37].

### Statistical analysis

The renal vasoconstrictor response to each agonist was taken as the mean of ascending and descending responses due to four doses. The comparison between the groups was based on the overall response calculated as the average of the four averaged responses. The data were presented as mean ± S.E.M. The statistical analysis for the renal vasoconstrictor studies was done by using one-way ANOVA followed by Bonferroni *post hoc* test for bar graph data of overall mean % drop in RCBP, while two-way ANOVA followed by Bonferroni *post hoc* test for dose response curves of renal vasoconstrictor study was performed using GraphPad Prism (GraphPad Software, Inc. CA, USA) with significance taken at P< 0.05. Gene expression data were analysed using the comparative method (ΔΔC_T_ method) and StepOne^™^ Software (Version 2.1, Applied Biosystem, USA).

## Results

### Effect of exogenous administered NaHS on SBP, MAP, heart and LV index and RCBP in control and LVH groups

**Fig 1:**
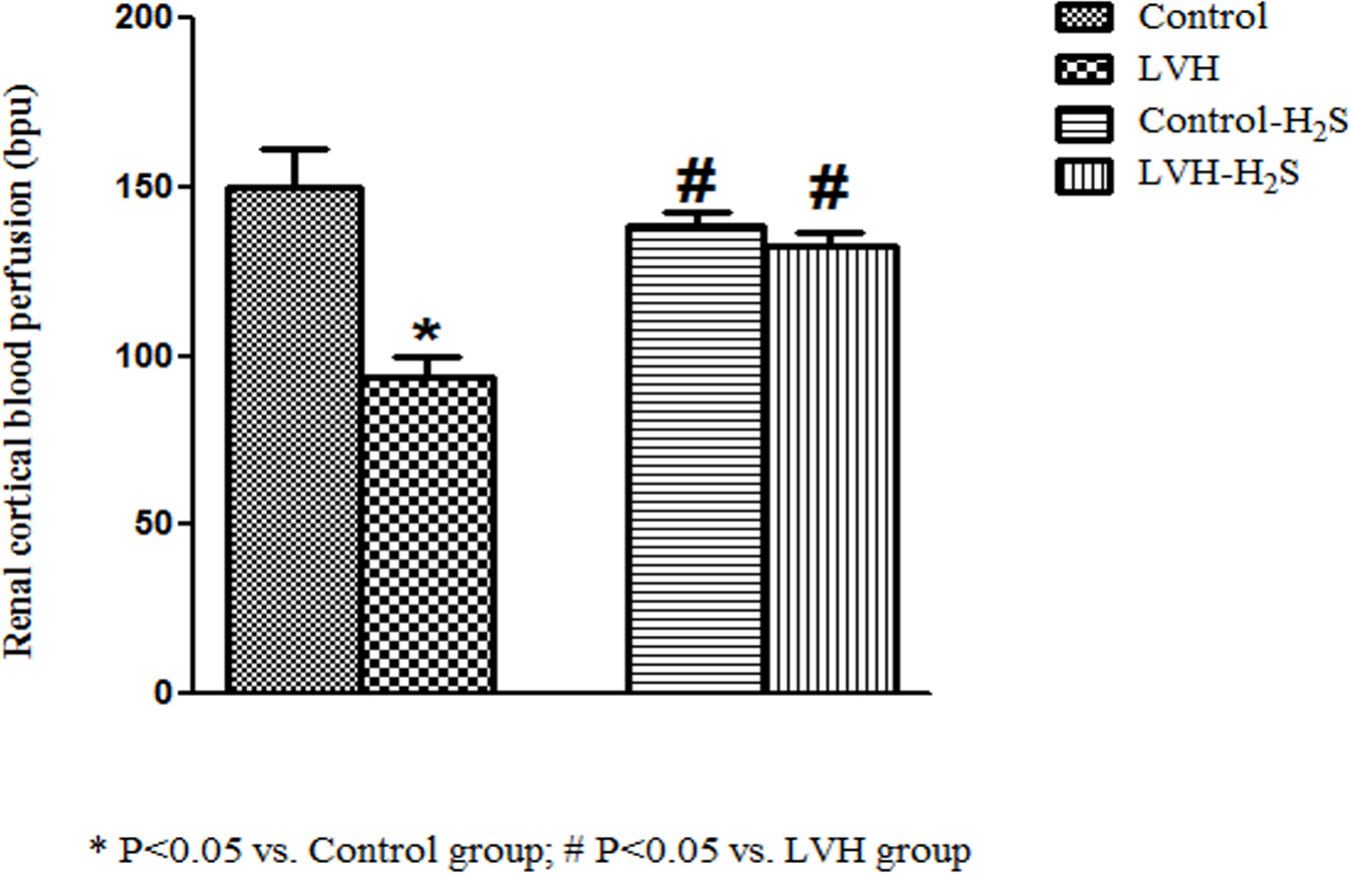
Renal cortical blood perfusion in Control, LVH, Control-H_2_S and LVH-H_2_S groups. Data are expressed as mean± SEM. * represents P<0.05 compared to Control while # represents P<0.05 compared to LVH group (n = 6).

SBP, MAP and LV index were significantly increased (P<0.05) in LVH when compared to Control group, while exogenous administration of H_2_S significantly (P<0.05) reduced them (Table 1). However, the RCBP in LVH group was lower (P<0.05) than control (LVH vs. control, 94±6 vs. 150±12 bpu). NaHS treatment in the LVH group resulted in a significant increase (P<0.05) in blood perfusion to the renal cortex compared to their untreated counterparts (132±5 vs. 94±6 bpu; Fig 1).

**Table 1:** The SBP, MAP heart index and LV index of control, LVH, control-H_2_S and LVH-H_2_S.

Parameters	Control	LVH	Control-H_2_S	LVH-H_2_S
SBP (mmHg)	132±4	159±5*	140±7#	135±2#
MAP (mmHg)	119±1	142±5*	122±6#	122±3#
LV index (%)	0.16±0.004	0.24±0.002*	0.19±0.006*#	0.21±0.001*#

All the data is expressed as mean± SEM.

* P<0.05 represents comparison with control group.

# P<0.05 represents comparison with LVH group.

SBP, systolic blood pressure; MAP, mean arterial pressure; LV, Left ventricle.

### H_2_S, NO and cGMP levels in kidney tissue and H_2_S in urine of NaHS treated and untreated LVH and controls

**Fig 2:**
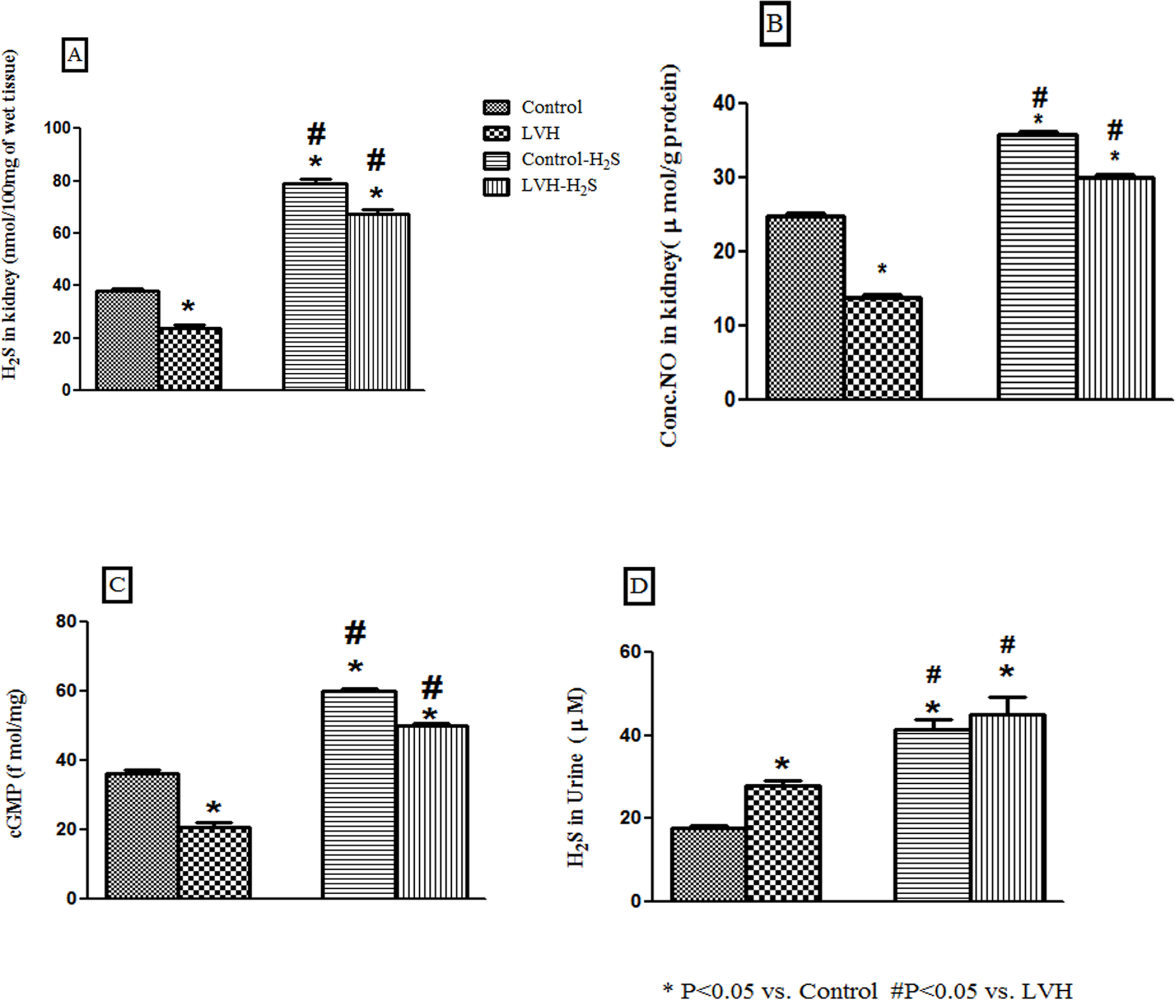
(A, B, C and D). Data showing the concentration of H_2_S (A) NO (B), cGMP (C) in the kidney and H_2_S in urine (D) of Control, LVH, Control-H_2_S and LVH-H_2_S groups. Data are expressed as mean± SEM. * represents P<0.05 compared to Control while # represents P<0.05 compared to LVH group (n = 6).

The concentration of H_2_S in the kidney tissue of LVH rats was significantly (P<0.05) lower than the control (LVH vs. control, 24±2 vs. 38±1nM/g of protein) but treatment of LVH rats with NaHS resulted in a significantly higher level of H_2_S compared to the untreated LVH rats (LVH-H_2_S vs. LVH,67±2 vs. 24±2 nM/g of protein; Fig 2A). Similarly, the concentration of NO of the kidney tissues in LVH rats was also lowered compared to the control (14±1 vs. 25±1μmol/g protein) but raised (22±1 μmol/g protein, P<0.05)following NaHS treatment compared to the untreated LVH rats (Fig 2B).

The cGMP levels of the kidney tissues in LVH rats was also lowered compared to the control (21±1 vs. 39±2fmol/mg protein), but it was higher in those LVH rats that were treated with NaHS (50±1 fmol/mg protein, P<0.05; Fig 2C).

The concentration of H_2_S in the urine of LVH rats was significantly (P<0.05) greater than the control (LVH vs. control, 18±1 vs. 28±1 μmol) but treatment of LVH rats with NaHS resulted in a significantly higher level of H_2_S in the urine as compared to the untreated LVH rats (LVH-H_2_S vs. LVH, 45±4 vs. 18±1μmol; Fig 2D).

### Relative CSE, CBS, 3-MST, eNOS expression and CSE activity in NaHS treated and untreated control and LVH rats

**Fig 3:**
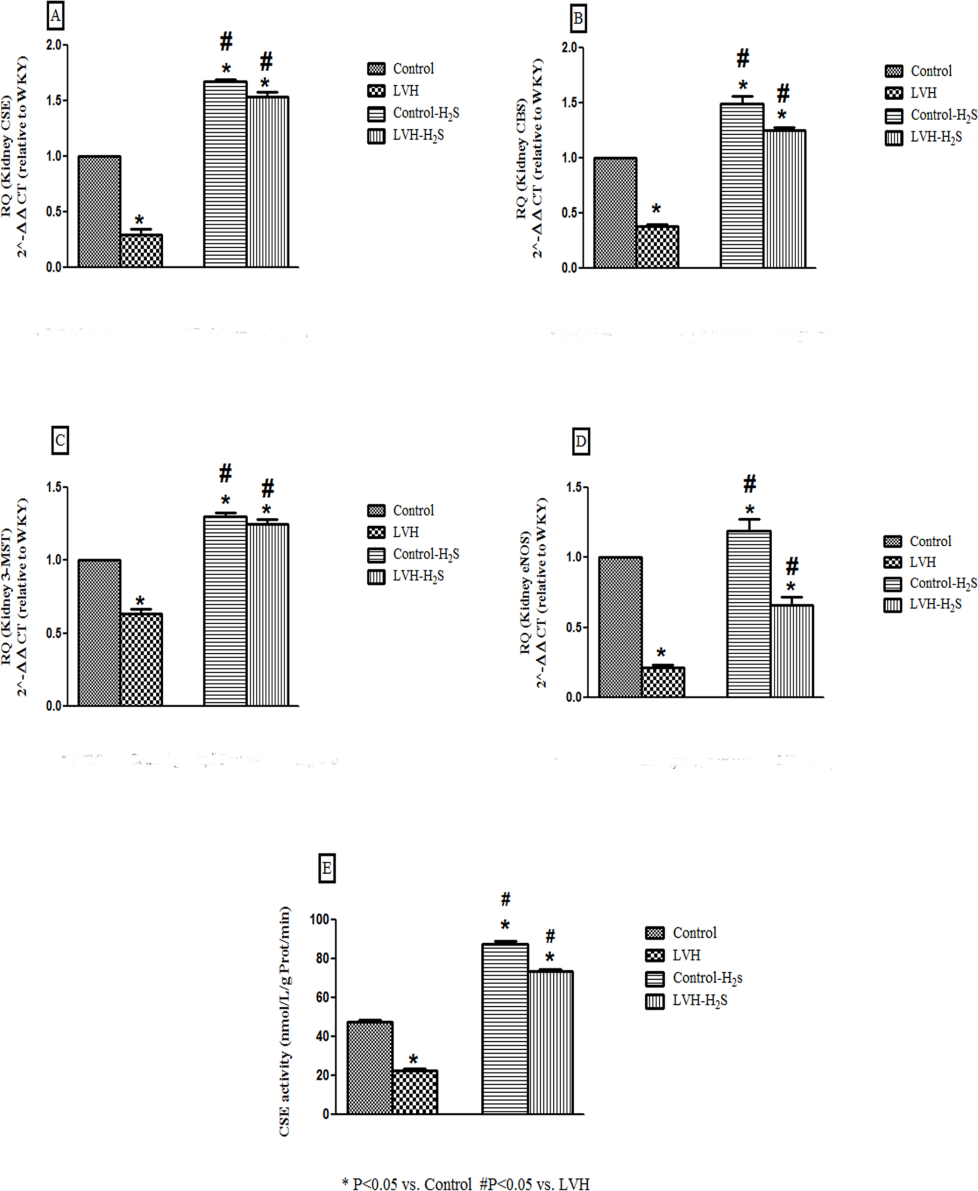
(A, B, C, D and E). Data showing the expression of CSE mRNA (A), CBS mRNA (B), 3-MST mRNA (C), eNOS mRNA (D) and CSE activity (E) in the kidney of Control, LVH, Control-H_2_S and LVH-H_2_S groups. Data are expressed as mean± SEM. * represents P<0.05 compared to Control while # represents P<0.05 compared to LVH group (n = 9 in triplicate).

LVH resulted in down regulation of CSE approximately74%, of CBS around 62%, of 3-MST approximately 37% and of eNOS by79% in the kidney when compared to CSE, CBS, 3-MSTand eNOS mRNA in the kidney of control rats. The treatment of control or LVH rats with NaHS resulted in upregulation of the CSE mRNA in the kidney by approximately 67% and 42.8% respectively (Fig 3A), CBS by approximately 49% and 22.9% (Fig 3B), and that of 3-MST expression by 30% and 98% respectively (Fig 3C), when compared to their untreated counterparts.

Upon NaHS treatment of control and LVH rats there was an upregulation of the eNOS mRNA in the kidney by approximately 46.6% and 21.4% respectively (Fig 3D). However, the CSE activity in the rat kidney of LVH group was significantly (P<0.05) reduced compared to CSE activity in the control group, while exogenous administration of H_2_S significantly increased (all P<0.05) CSE activity in the kidney of control-H_2_S and LVH-H_2_S (CSE activity (nmol/L/g Prot/min); control: 48±1; LVH: 23±1; control-H_2_S: 88±1 and LVH-H_2_S: 74±1; Fig 3E).

### Vasoconstrictor responses

#### α_1A_-adrenoceptor subtype responses to adrenergic agonists

**Fig 4:**
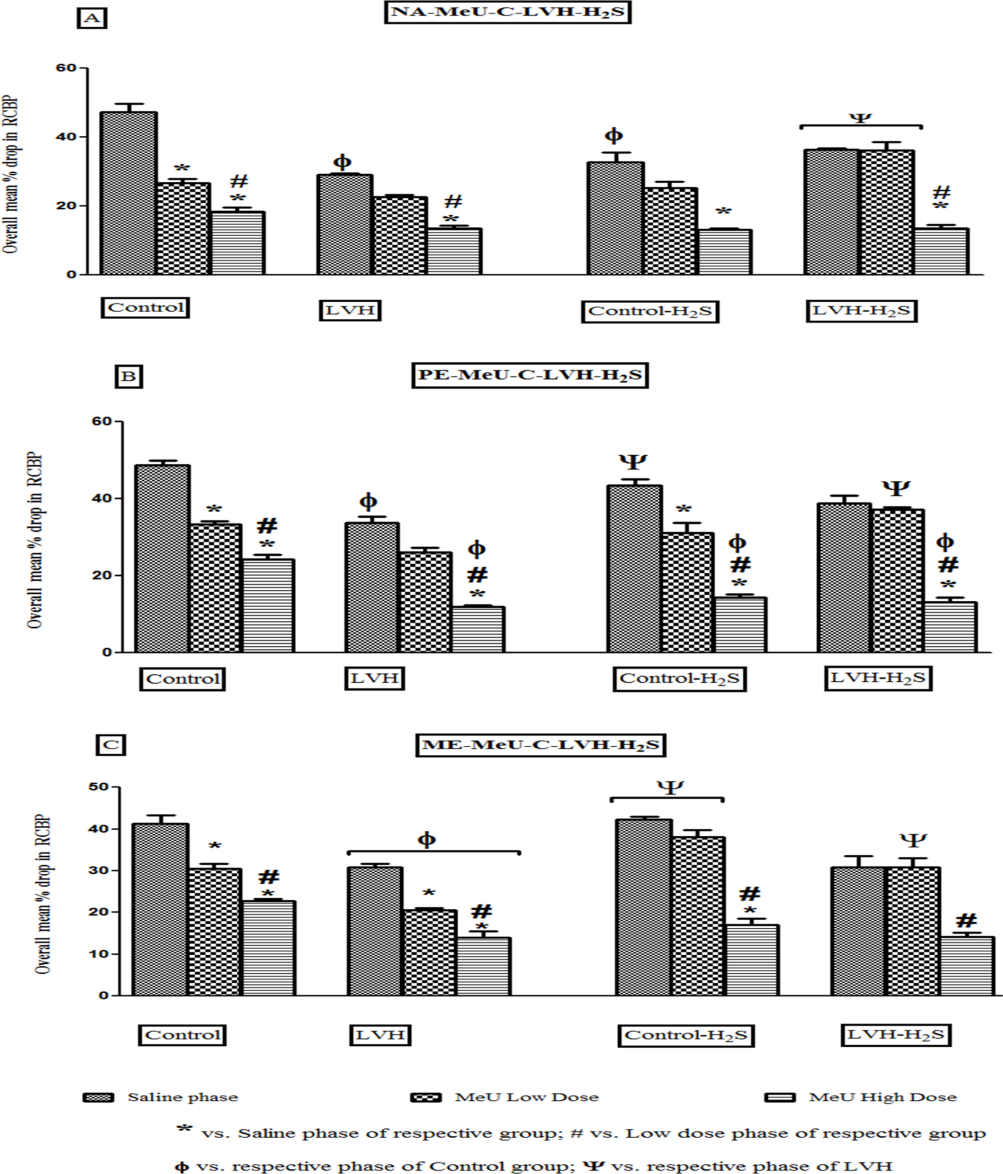
(A, B and C). Bar graph showing the overall mean of % drop in renal cortical blood perfusion in response to NA (A), PE (B) and ME (C) in Control, LVH, Control-H_2_S and LVH-H_2_S groups rats during saline, 5-MeU low dose and 5-MeU high dose phases. Values are mean± SEM of n = 6 rats in each group. * P<0.05 vs. Saline phase of same group and # P<0.05 vs. 5-MeU low dose phase of same group. ϕ P<0.05 vs. respective phase of Control and Ψ P<0.05 vs. respective phase of LVH groups.

The magnitude of the renal vasoconstrictor responses to NA and ME but not PE in LVH rats were significantly (all P<0.05) blunted compared to their control counterparts (LVH vs. Control, NA; 30±1 vs. 45±3%, ME; 32±1 vs. 44±2%). The exogenous administration of NaHS resulted in augmented vasoconstrictor responses to NA but not to ME or PE (LVH-H_2_S vs. LVH, 36±1 vs. 28±1%; Fig 4A, 4B & 4C).

**Fig 5:**
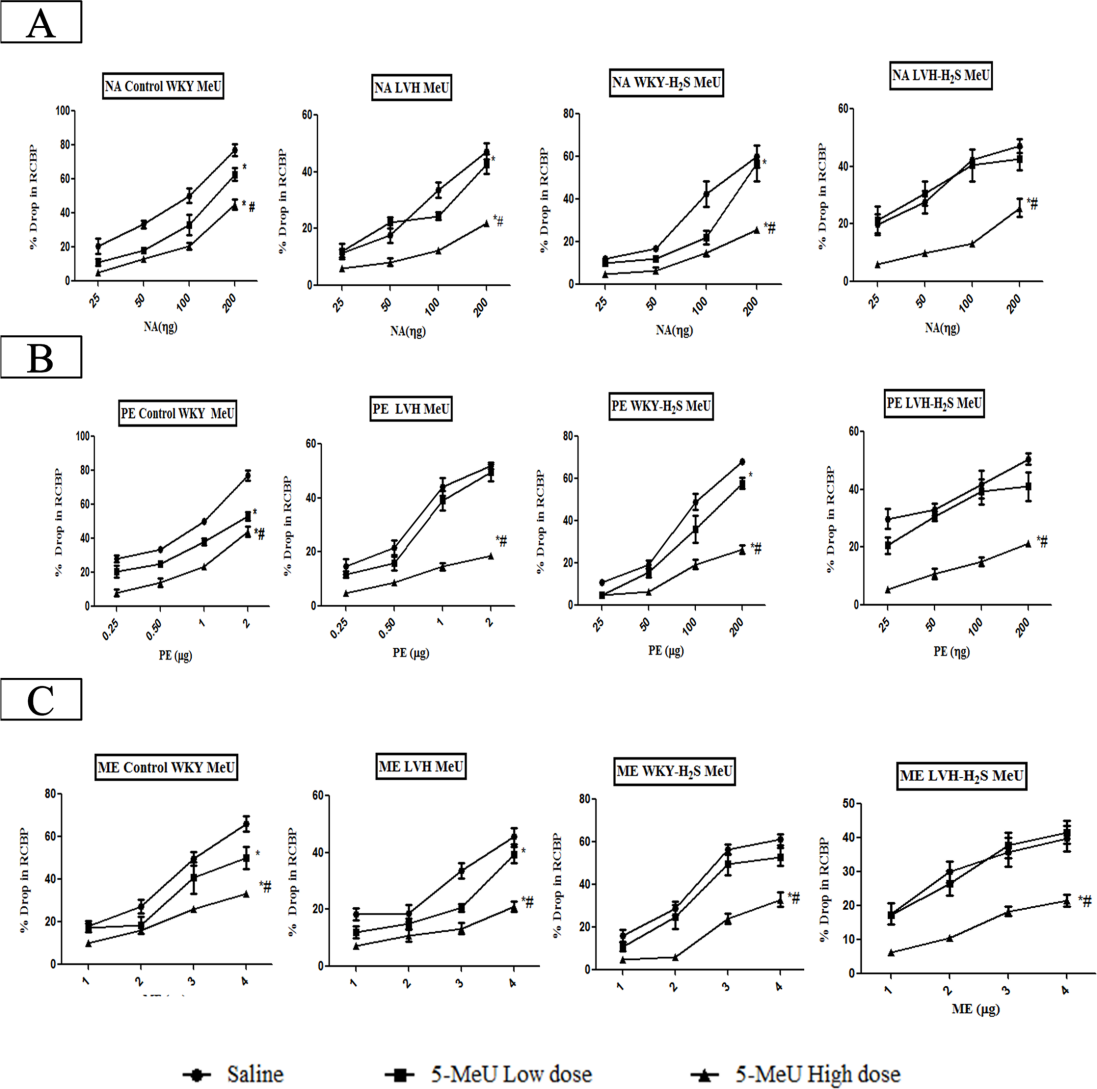
(A, B and C). Dose response curve of renal vasoconstriction responses to set of doses of NA (A), PE (B) and ME (C) in Control, LVH, Control-H_2_S and LVH-H_2_S groups rats during saline phase, low dose phase and high dose phase of 5-MeU. Values are mean± SEM of n = 5–7 rats in each group. The significance is overall mean of 4 graded doses (each dose response is averaging the ascending and descending order responses) of an agonist in each phase and compared to saline phase and high dose phase. * P<0.05 vs. Saline phase and # P<0.05 vs. 5-MeU low dose phase.

The renal vasoconstrictor responses to NA in the saline phase in the LVH-H_2_S group were significantly (P<0.05) increased by 29% when compared to LVH group. Following blockade of the α_1A_-adrenoceptor with low doses of 5-MeU, the renal vasoconstriction elicited by α_1A_-adrenoceptor activation by exogenous administration of NA in the LVH-H_2_S group was significantly (P<0.05) increased by 44% when compared to that in the LVH group. Blocking the α_1A_-adrenoceptor with the high dose of 5-MeU, caused the response elicited by α_1A_-adrenoceptor with exogenous administration of NA in the LVH-H_2_S group to be increased by 17% when compared to that in the LVH group but the magnitude of these responses was not significantly different (Figs 4A & 5A). The dose response curves of different doses of NA in Control, LVH, Control-H_2_S and LVH-H_2_S in the absence and presence of 5-MeU are shown in Fig 5A.

Induction of LVH significantly (P<0.05) reduced the renal vascular responses to the α_1A_-adrenoceptoragonist PE in the saline phase by 38% when compared to those obtained to PE in the saline phase of the control group. The exogenous administration of H_2_S to LVH augmented the renal vascular responses to PE, the α_1A_-adrenoceptor agonist, in the saline phase by 22%. Blocking α_1A_-adrenoceptorsusing low doses of 5-MeU, caused the renal vasoconstrictor responses elicited by the exogenous administration of PE to the LVH-H_2_S group to be significantly (P<0.05) increased by 42% when compared to those obtained in the LVH group. By contrast, there was no significant increase in renal vascoconstrictor responses to PE when the α_1A_-adrenoceptor was blocked with the high doses of 5-MeU (Figs 4B and 5B). The dose response curves of different doses of PE in Control, LVH, Control-H_2_S and LVH-H_2_S in the absence and presence of 5-MeU are shown in Fig 5B.

Induction of LVH significantly (P<0.05) reduced renal cortical blood perfusion when ME was given in the saline phase by 41%, when compared to those obtained in the saline phase of control group, while exogenous administration of H_2_S had no effect on responses to ME in saline phase of LVH. Blocking the α_1A_-adrenoceptor using low doses of 5-MeU, significantly (P<0.05) increased the renal vasoconstrictor responses elicited by ME by 41% in the LVH-H_2_S group when compared to the LVH group during the low dose phase. There was no significant change in the magnitude of the renal vascular responses to ME when the adrenoreceptor was blocked with high doses of 5-MeU Fig 4C. The dose response curves of different doses of ME in Control, LVH, Control-H_2_S and LVH-H_2_S in the absence and presence of 5-MeU are shown in Fig 5C).

#### α_1B_-adrenoceptor subtype responses to adrenergic agonists

**Fig 6:**
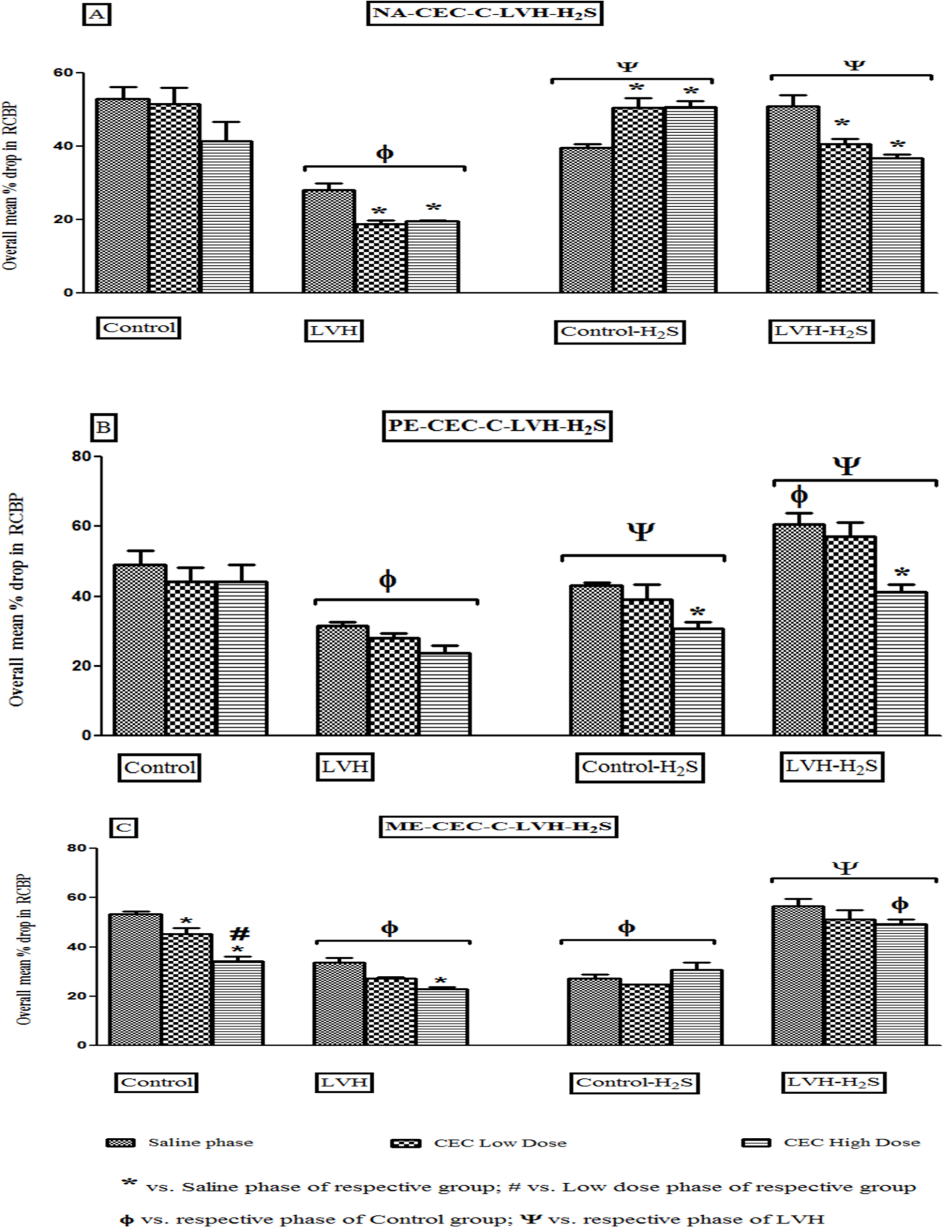
(A, B and C). Bar graph showing the overall mean of % drop in renal cortical blood perfusion in response to NA (A), PE (B) and ME (C) in Control, LVH, Control-H_2_S and LVH-H_2_S groups rats during saline, CEC low dose and CEC high dose phases. Values are mean± SEM of n = 6 rats in each group. * P<0.05 vs. Saline phase of same group and # P<0.05 vs. CEC low dose phase of same group. ϕ P<0.05 vs. respective phase of Control and Ψ P<0.05 vs. respective phase of LVH groups.

**Fig 7:**
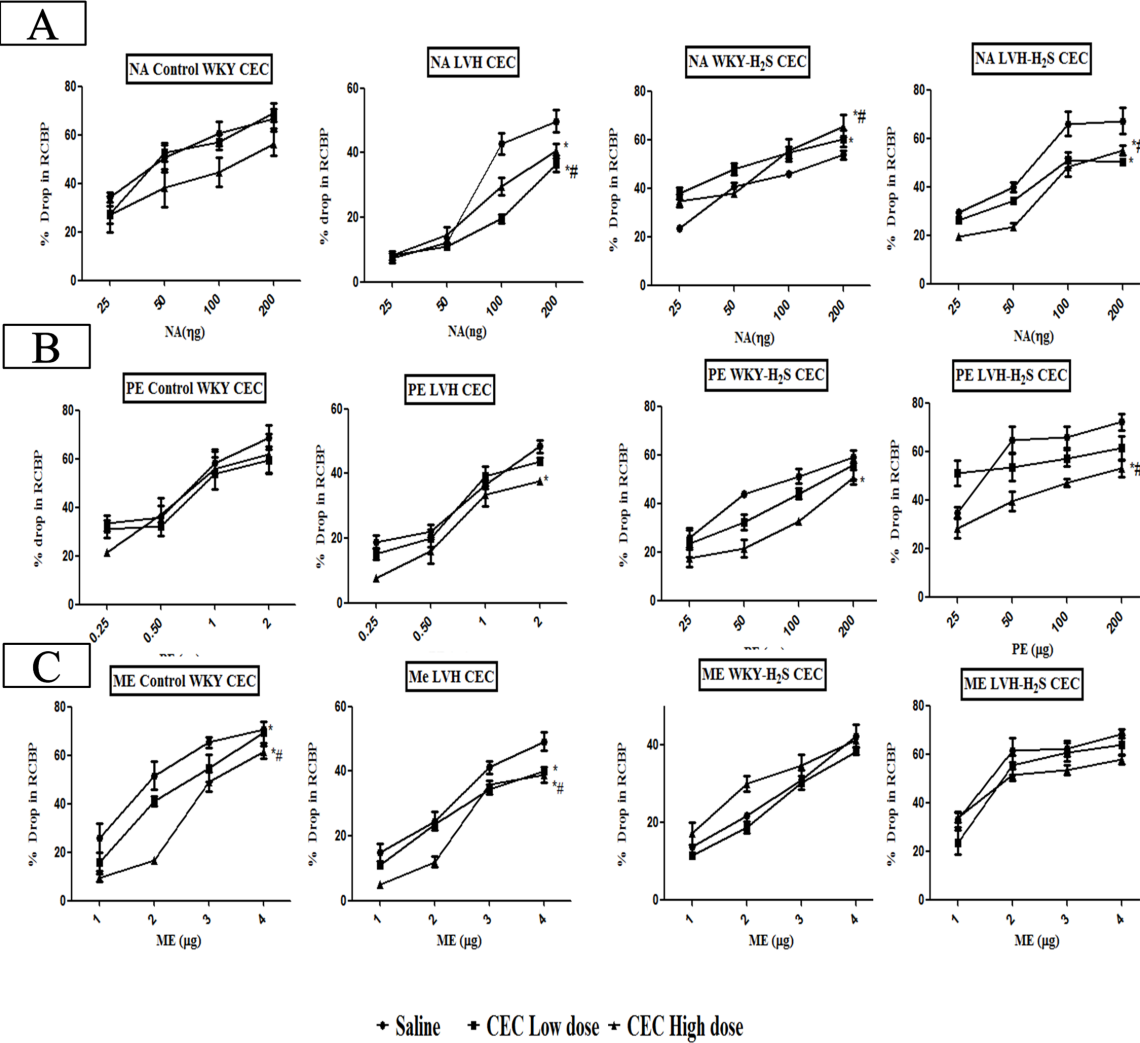
(A, B and C). Dose response curve of renal vasoconstriction responses to set of doses of NA (A), PE (B) and ME (C) in Control, LVH, Control-H_2_S and LVH-H_2_S groups rats during saline phase, low dose phase and high dose phase of CEC. Values are mean± SEM of n = 5–7 rats in each group. The significance is overall mean of 4 graded doses (each dose response is averaging the ascending and descending order responses) of an agonist in each phase and compared to saline phase and high dose phase. * P<0.05 vs. Saline phase and # P<0.05 vs. CEC low dose phase.

Induction of LVH significantly (P<0.05) reduced the renal vasoconstrictor responses to NA in the saline phase by 35% when compared to responses to NA in saline phase of control group. The renal vasoconstrictor responses to NA in the saline phase of LVH-H_2_S were significantly (P<0.05) increased by 82% when compared to the LVH group. Blocking the α_1B_-adrenoceptor using low doses of CEC, caused the renal vascular responses elicited by NA in the LVH-H_2_S group to be significantly (P<0.05) increased by 11.6% when compared to those produced by NA in the LVH group. Blocking the α_1B_-adrenoceptor using high doses of CEC, significantly (P<0.05) increased the renal vascular responses to exogenous NA by 85% compared to those obtained in the LVH group although this was not significant when compared to the administration of NA in the control group (Fig 6A). This showed that exogenous administration of H_2_S in LVH group significantly (P<0.05) increased the renal vascular responses produced by NA in the saline, low and high dose phases of antagonists when compared to responses to NA in saline, low dose and high dose phases of antagonists in the LVH group Fig 6A while the dose response curves of different doses of NA in control, LVH, control-H_2_S and LVH-H_2_S in the absence and presence of CEC are shown in Fig 7A.

Induction of LVH significantly (P<0.05) reduced the renal vascular responses PE in the saline phase by 47% when compared to control group. The renal vasoconstrictor responses to PE in saline phase of LVH-H_2_S were significantly (P<0.05) increased by 91% when the same responses were compared in the LVH group. Blocking the α_1B_-adrenoceptor with low doses of CEC, the renal vascular responses elicited by PE in the LVH-H_2_S group were significantly (P<0.05) increased by 10.4% when compared to LVH group. Moreover, when the high doses of CEC were given, the renal vasoconstrictor responses PE in LVH-H_2_S group were increased significantly (P<0.05) by 71% compared to those produced by PE in the LVH group (Fig 5B). This showed that exogenous administration of H_2_S in the LVH group significantly (P<0.05) enhanced the renal vasoconstrictor responses produced by the α_1B_-adrenoceptor to PE in the saline, low dose and high dose phases of antagonist when compared to responses produced to PE in saline, low dose and high dose phases of antagonists in LVH rats (Fig 6B). Dose response curves of different doses of PE in Control, LVH, Control-H_2_S and LVH-H_2_S in the absence and presence of CEC are shown in Fig 7B.

Induction of LVH significantly (P<0.05) reduced the renal vascular responses to ME in the saline phase by 36% compared to those obtained in saline phase of the corresponding control group. The renal vasoconstrictor responses to ME in saline phase of LVH-H_2_S were significantly (P<0.05) increased by 73% when compared to those obtained in the LVH group. Blocking the α_1B_-adrenoceptor using the low doses of CEC, significantly (P<0.05) increased by 89% the renal vasoconstrictor responses elicited by ME in LVH-H_2_S group compared to those obtained in the LVH group. During the high doses of CEC, the renal vasoconstrictor responses to ME in the LVH-H_2_S group were increased significantly (P<0.05) by 11.3% compared to those produced by ME in the LVH group (Fig 5C). This showed that exogenous administration of H_2_S in the LVH group significantly (P<0.05) increased the renal vascular responses produced by α_1B_-adrenoceptor activation by ME in the saline low dose and high dose phases of antagonist when compared to those to ME in same phases of the LVH group (Fig 6C). The dose response curves of different doses of ME in Control, LVH, Control-H_2_S and LVH-H_2_S in the absence and presence of CEC are shown in Fig 7C.

### Histopathological evidences

**Fig 8:**
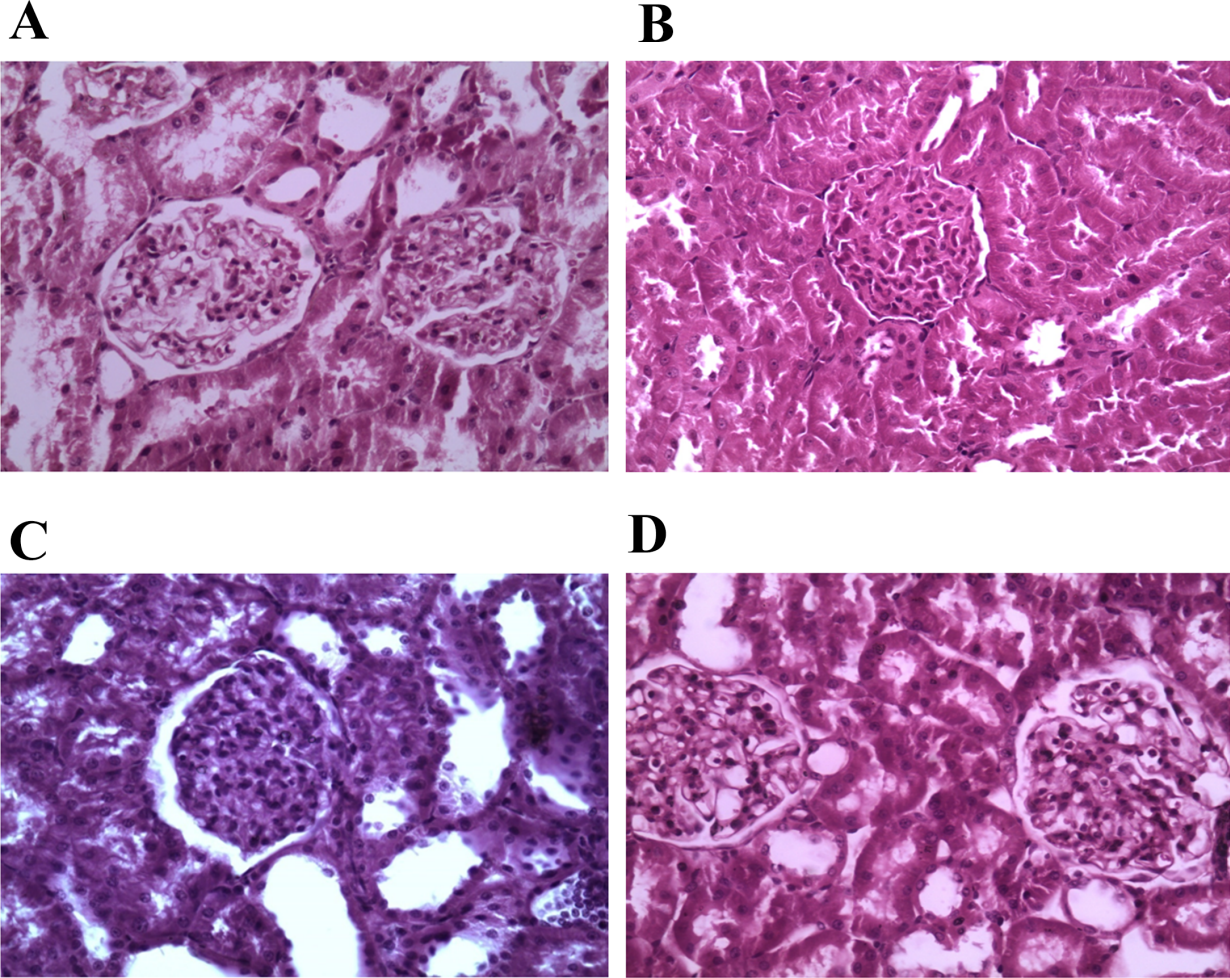
Histopathological evidence of rat kidney of Control, LVH, Control-H_2_S and LVH-H_2_S groups using hematoxyllin and eosine staining.

Histopathological examination of kidney did not show any abnormality related to tubules and glomerulus, in addition there were no signs of inflammation, hyaline cast or fibrosis in LVH-WKY groups (Fig 8A–8D).

## Discussion

The present study was based on investigation of the interaction between the gasotransmitter H_2_S and α_1A_ and α_1B_-adrenoceptors in the renal vasculature in LVH rat model and also explored the hypothesis that (*i*) exogenous administration of H_2_S would regulate the eNOS/NO/cGMP pathway in the kidney which in synergism with H_2_S could also increase the renal vascular sensitivity to α_1A_ and α_1B_-adrenoceptor activation in the LVH rats; (*ii*) whether exogenous administration of H_2_S would interact and upregulate the eNOS/NO/cGMP pathway in control and disease conditions. The first major finding was an upregulation of the CSE/H_2_S pathway in the kidney following exogenous administration of NaHS. This was associated with an increased sensitivity of the renal vasculature to α_1A_ and α_1B_-adrenoceptors activation as in the in LVH rats, the sensitivity of these agonists was blunted. Treatment of LVH rats with NaHS resulted in enhancement of not only the CSE/H_2_S pathway but also modulated the eNOS/NO/cGMP pathway which was associated with an increased sensitivity of the renal vascular α_1A_ and α_1B_-adrenoceptors to exogenously administered adrenergic agonists.

Increased SBP, MAP and LV index in response to model induction in present study is in line with our previous study on this model [52]. Exogenous administration of H_2_S significantly restored the manifestation of LVH pointing out antihypertrophic role of H_2_S which has also been recently reported [36]. The exogenous administration of NaHS in LVH rats increased the blunted renal cortical blood perfusion. This is similar to the findings in spontaneously hypertensive rats (SHR) whereby exogenous H_2_S donor increased the baseline renal cortical blood perfusion [53]. It is possible that in this model, the lower renal cortical blood perfusion in LVH may be due to an increased local vasoconstriction within the kidney due to the elevated circulating noradrenaline as well as angiotensin II levels [23,54–56], or it may be the local action of the H_2_S in the cortex of kidney of LVH rats treated with H_2_S. It is also possible that the vasodilator effect of H_2_S could offset the vasoconstrictor action of both factors and reduce the increased vascular tone in the kidney. This notion is supported by a previous study which demonstrated that exogenous administration of H_2_S resulted in greater pre-glomerulus arteriolar vasodilation and resulted in increased GFR and renal blood flow [22]. Local vasodilation by H_2_S in the kidney can be evidenced by the upregulation of CSE, CBS and 3-MST mRNAs in the renal cortex of NaHS treated LVH rats. In addition it was also surprising that exogenous administration of H_2_S donor significantly increased the expression of CSE, CBS and 3-MST mRNAs in the renal cortex which is in accordance with recently reported data [19]. The up-regulation of CSE, CBS and 3-MST expression in the cortex pointed towards the augmented local production of H_2_S, which may have negative impact on the CSE activity as reported [57]. While investigating CSE/H_2_S pathway in the kidney in the present study, it was observed that upon increased CSE activity in the kidney, there was also a corresponding increase in H_2_S concentration, thus indicating H_2_S production in the kidney. The elevated H_2_S levels may cause poisoning in brain, however elevated H_2_S levels in the urine indicated that there is no H_2_S accumulation in the plasma. Estimation of thiosulphate level would have resolved the possibility of H_2_S toxicity [58], but this was the limitation of the present study. However, it is possible that this upregulation of CSE/H_2_S in the kidney may reduce renal vascular tone via vasodilator pathways which modify the functional behaviour of α_1_-adrenoceptors which are desensitized in LVH model [23]. Therefore, it can be deduced that enhancement in renal cortical blood perfusion in LVH-H_2_S is due to augmented CSE expression in the cortex along with upregulated CBS and 3-MST, increased CSE activity and corresponding increase in H_2_S concentration in the kidney.

The magnitude of the renal vasoconstrictor responses to NA, PE and ME was lower in the saline or pre-drug phase of LVH when compared to those produced in the control group in the present study. It therefore indicated a possible modulation of α_1A_ adrenoreceptor’s function in the kidney of LVH, which is in accordance with previous studies on α_1_ adrenoreceptor’s function in the kidney of LVH rats [23,24]. The blunted response to adrenergic agonists observed in LVH in the present study is associated with a down regulation of CSE/H_2_S and eNOS/NO pathways in the kidney of such animal models [23]. The up-regulation of CSE/H_2_S pathway in the kidney of LVH rats upon exogenous administration of the substrate NaHS, an H_2_S donor and consequent augmentation of the renal vascular responses to NA, PE and ME, indicated an action of H_2_S to produce a vasodilation or reduction in vascular tone which was potent enough to modulate the vasoconstrictor responses. This however was the case when α_1A_-adrenoreceptors are partially blocked. These augmented responses showed a possible interaction between H_2_S and α_1A_ adrenoreceptors which may exist in the renal vasculature but the exact mechanism of action is still unclear, however possibly it is due to the modification of G-protein coupled 2nd messenger pathway or up regulation of α_1A_ and α_1B_-adrenoreceptors.

The present findings supported the view that there could be a shift in the functional contribution of the α_1A_-adrenoreceptor subtype towards the α_1B_-adrenoreceptor subtype which was also observed in previous studies using the same LVH model [23]. In addition increased responsiveness of α_1B_-adrenoreceptors to NA, PE and ME may be attributed to the local vasodilation in the kidney by the upregulation of CSE/H_2_S and eNOS/NO/cGMP pathways, which is supported by a previous study in which α_1B_-adrenoreceptor subtype mediated the renal vasoconstriction in a rat model of chronic renal disease induced with cisplatin [59]. The interaction between H_2_S and α_1B_-adrenoreceptors is unknown, but it might be explained by a potentiating effect of H_2_S on the responsiveness of these receptors to NA, PE and ME in present model of LVH by modulation of the eNOS/NO/cGMP pathways. This notion is supported by the fact that H_2_S stimulated vasodilatation are dependent on _C_GMP [60]. Moreover, it is expected that up regulated eNOS/NO/cGMP pathways would reduce the vascular tone by vasodilation in the kidney. Another possible reason for the augmented responses to α_1B_-adrenoreceptor activation in the LVH-H_2_S group may be the buffering effect of H_2_S against elevated levels of the vasoconstrictor angiotensin II [61] as H_2_S has ACE inhibitor activity[62]. This enhanced responsiveness could also be attributed to the increased expression of CSE mRNA in the kidney cortex observed in present study which could ultimately lead to an elevated regional concentration of H_2_S.

**Fig 9:**
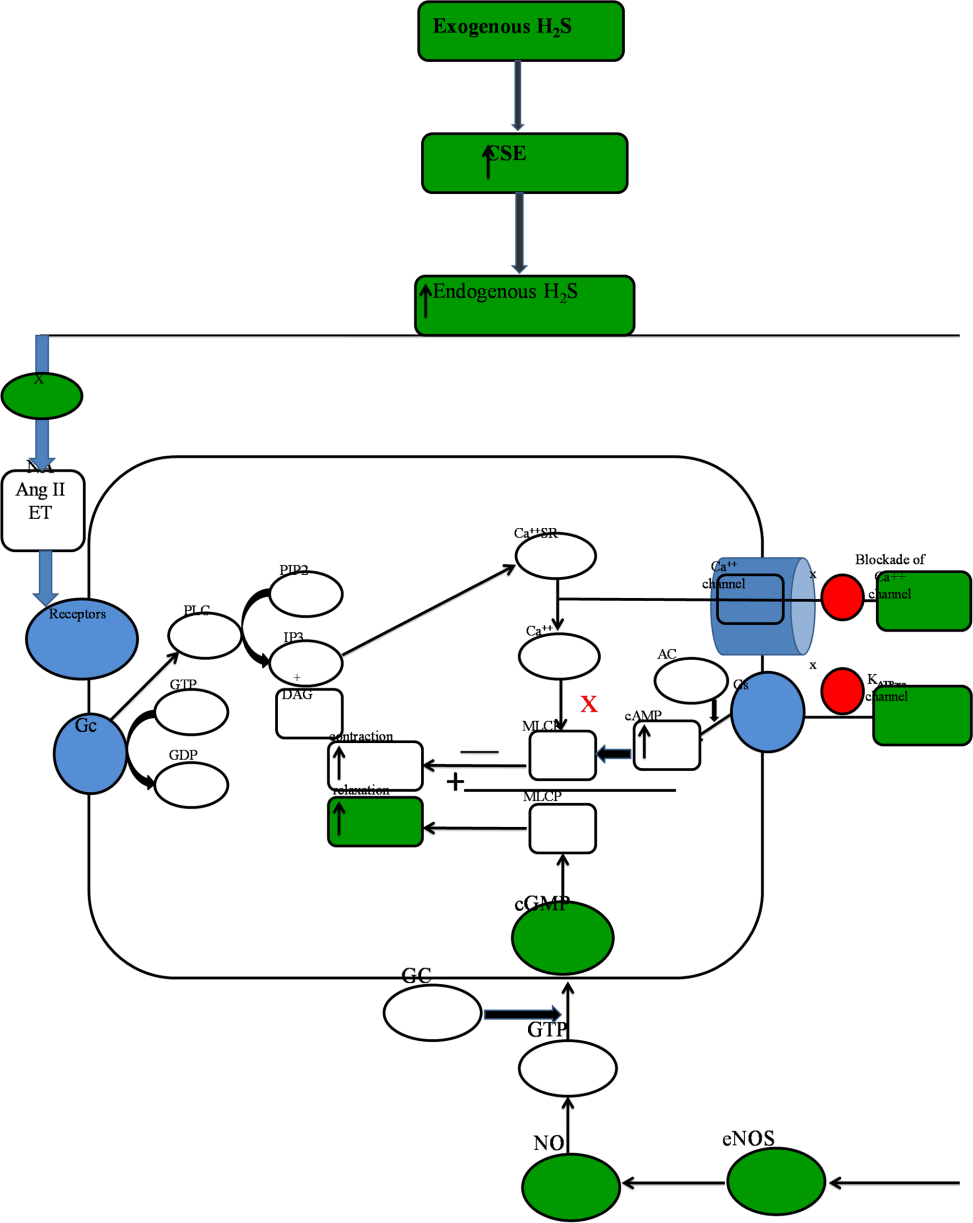
Mechanism of action of hydrogen sulphide in resensitization of α_1_- adrenoreceptors by modifying the G-protein coupled 2nd messenger pathway.

There are few possibilities for decrease in vasoconstriction responses of α_1_ adrenoreceptors in LVH which may be due to either down regulation of CSE and eNOS mRNAs expression [24], desensitization of the receptors [6,63] or alteration in G-protein system due to hyperactivity of the sympathetic nervous system. This hyperactive sympathetic nervous system elicits physiological responses mediated by G-protein coupled adrenergic receptors [64] which use a guanylyl cyclase pathway. The blunt responses of α_1A_ and α_1B_-adrenoreceptors upon activation by NA, PE and ME in LVH may be explained on the basis of increased vascular tone in the kidney due to continuous exposure to vasoconstriction, down regulation of vasodilator pathways, modification of G-protein coupled 2^nd^ messenger pathway system and reduced expression of adrenergic receptors in the kidney. The present study also evaluated the vasodilator CSE/H_2_S and eNOS/NO/cGMP pathways in the kidney and demonstrated that exogenous administration of NaHS as a substrate up-regulated the CSE/H_2_S pathway in the kidney. The novel finding of the present study is the modulation of the eNOS/NO pathways in both normal and disease state, where the increased eNOS/NO levels in the kidney indicated that exogenous administration of NaHS result in an up-regulation of the eNOS/NO pathway which being a vasodilator pathway could reduce vascular tone in the kidney. These findings can be vindicated by other study that showed that induction of LVH by the administration of isoprenaline and caffeine result in an increased plasma concentration of vasoconstrictors noradrenaline and angiotensin II levels and down regulation of CSE mRNA in the heart [37]. Up-regulation of CSE mRNA expression in the kidney can offset the responses produced by these vasoconstrictors which may be the contributory factor for reduced responsiveness of α_1_-adrenoreceptors. The present study also showed that H_2_S donor not only up-regulated CSE/H_2_S pathway in the kidney but also up-regulated other H_2_S producing enzymes like CBS and 3-MST. These up-regulated H_2_S producing enzymes are expected to overcome the vasoconstriction being induced by noradrenaline and angiotensin II as reported in previous study [37], and increased the responsiveness of α_1A_ and α_1B_ adrenoreceptors in the kidney. The increased CSE activity in the kidney in LVH-H_2_S group indicated the significance of interaction between H_2_S and responsiveness of α_1_-adrenoreceptors subtypes, which had also been observed previously where blunted response to adrenergic agonists in LVH was associated with a down regulation of CSE/H_2_S and eNOS/NO pathways in the kidney of these animal models [23]. The exact way by which CSE/H_2_S axis reduced the vascular tone in the kidney is not known yet but it might be due to the activation of ATP-sensitive potassium channels [26]. The K_ATP_ channels are physiologically the primary target for adenylate cyclase/cAMP/protein kinase A signalling pathway [65] and this ATP-sensitive potassium channel may cause vasorelaxation by increasing the cAMP pathway [66] (Fig 9). In the present study when we investigated whether cGMP levels were elevated in the kidney tissue of LVH group treated with H_2_S compared to control which would be consistent with the upregulation of eNOS/NO/cGMP pathway. The observation that in LVH cGMP levels was increased following NaHS is consistent with previous studies which reported the impact of exogenous administration of H_2_S which increased the CGMP pathway by inhibiting Phosphodiestrase [60,67]. These findings support a previously reported study which showed CSE/H_2_S mediated vasodilation and smooth muscle relaxation via a cyclic guanylyl monophosphate pathway in an independent manner [12] being operated by a G-protein coupled second messenger pathway system. This modulation of the G-protein system may be one of the reasons for augmentation of the responses of α_1_ adrenoreceptor activation which are operated via G-protein[64]. The increased cGMP mediated by NO due to exogenous administration of NaHS and H_2_S generation also prevents the degradation of cGMP by inhibiting the phosphodiestarases 5 (PDE-5) [68]. This up-regulation of eNOS/NO/cGMP pathway in synergy with CSE/H_2_S would not only increase the vasodilation but also modify the G-protein coupled 2^nd^ messenger pathway system responsible for augmented responsiveness of α_1A_ and α_1B_-adrenoreceptors. The increased NO production following exogenous administration of NaHS and H_2_S has been the subject of investigation over the last decade [26,29,69]. We have shown from molecular and *ex-vivo* evidence that exogenous administration of NaHS to increase H_2_S endogenously upregulated the eNOS/NO/cGMP pathways in the kidney of normal and LVH rats. However, the exact mechanism by which H_2_S augmented the responses of α_1A_ and α_1B_-adrenoreceptors to these adrenergic agonists could not be defined but it may possibly be explained on the basis of the upregulation of CSE/H_2_S and eNOS/NO/cGMP pathways in the kidney. Furthermore, exogenous administration of NaHS to increase endogenous H_2_S modulates the eNOS/NO/cGMP pathways in the kidney in both normal and disease states. However, future work is required on the expression of these α_1A_ and α_1B_-adrenoreceptors mRNAs to observe whether exogenous administration merely improved the responsiveness of these adrenoreceptors or whether there is up-regulation of respective mRNAs.

## Conclusion

In conclusion, in the present study the treatment of LVH with H_2_S resulted in up-regulation of CSE/H_2_S pathway, increased CSE activity and eNOS/NO/cGMP pathways in the kidney. These up-regulations of CSE/H_2_S and eNOS/NO/cGMP pathways enhanced the responsiveness of α_1A_ and α_1B_-adrenoreceptors subtypes to adrenergic agonists in LVH-H_2_S. These findings indicate an important role of H_2_S in modulating deranged signalling in the renal vasculature resulting from the development of LVH.
